# A primer on selecting grain boundary sets for comparison of interfacial fracture properties in molecular dynamics simulations

**DOI:** 10.1038/s41598-017-08637-z

**Published:** 2017-08-21

**Authors:** Rémi Dingreville, Doruk Aksoy, Douglas E. Spearot

**Affiliations:** 1 Sandia National Laboratories, Albuquerque, NM USA; 2 Department of Mechanical & Aerospace Engineering, University of Florida, Gainesville, FL USA

## Abstract

All grain boundaries are not equal in their predisposition for fracture due to the complex coupling between lattice geometry, interfacial structure, and mechanical properties. The ability to understand these relationships is crucial to engineer materials resilient to grain boundary fracture. Here, a methodology is presented to isolate the role of grain boundary structure on interfacial fracture properties, such as the tensile strength and work of separation, using atomistic simulations. Instead of constructing sets of grain boundary models within the misorientation/structure space by simply varying the misorientation angle around a fixed misorientation axis, the proposed method creates sets of grain boundary models by means of isocurves associated with important fracture-related properties of the adjoining lattices. Such properties may include anisotropic elastic moduli, the Schmid factor for primary slip, and the propensity for simultaneous slip on multiple slip systems. This approach eliminates the effect of lattice properties from the comparative analysis of interfacial fracture properties and thus enables the identification of structure-property relationships for grain boundaries. As an example, this methodology is implemented to study crack propagation along Ni grain boundaries. Segregated H is used as a means to emphasize differences in the selected grain boundary structures while keeping lattice properties fixed.

## Introduction

Grain and phase boundaries are key microstructural elements that play an important role in the confinement of local heterogeneous deformation processes, often leading to crack initiation followed by crack propagation along the interface planes. Of course, all grain boundaries are not equal in their innate predisposition for fracture. Experimental evidence in the field of grain boundary engineering indicates that the fundamentals of fracture processes in polycrystals are strongly influenced by grain boundary structure and its coupling with deformation processes^1–3^. Specifically, not only does the orientation relationship between the adjacent lattices and the orientation of the boundary plane affect boundary brittleness, but the interfacial structure is also an important contributing factor^4, 5^. In the case of grain boundaries, it is often assumed that “special” grain boundaries with low coincidence site lattice (CSL) designations should improve a material’s resistance to intergranular fracture^6–8^ because of the high density of coincident lattice sites. For example, experimental characterization in tungsten^9^ showed that special boundaries, such as Σ3 and Σ9 grain boundaries, possess higher resistance to failure in comparison with other types of CSL and random boundaries. However, CSL designation does not necessarily correlate with improved interfacial properties.

The analytical treatment of interfacial fracture considers the dependence on crystallography through both the surface energy of the boundary and the elastic mismatch between the two adjacent materials (i.e., Dundurs’ parameters used in continuum fracture mechanics^10^). However, it neglects the underlying atomic structure of the interface. At the atomic scale, many atomistic simulation studies have sought to not only extract the strength and/or the work of separation of individual grain boundaries^11–14^ but also provide fundamental insights on the crack tip fracture mechanisms^15–19^. Most of these atomistic studies explore grain boundary fracture for a limited class of grain boundaries, namely symmetric tilt and twist grain boundaries. Indeed, it is common in atomistic simulations for a range of grain boundary structures to be studied within the misorientation/structure space by incrementing the misorientation angle between minimum and maximum bounds around a fixed misorientation axis^16^ (e.g., symmetric tilt grain boundaries between 0° and 90° around the [001] axis). Databases considering general studies of the energies and structures of various boundaries exist^20, 21^. However, any comparative analysis of the mechanical behavior of boundaries within the misorientation space in these types of datasets becomes tenous since a change in the misorientation induces both a change in the interface structure and in the mechanical properties of the adjoining lattices simultaneously. For example, Spearot *et al*.^22^ illustrated this argument by showing that the maximum tensile strength of symmetric tilt bicrystal grain boundary models is predominantly influenced by lattice orientation, and is less sensitive to the grain boundary structure. Although there are some examples where grain boundary structure plays a commanding role^23^, in general, decoupling the role of the interface structure itself from lattice heterogeneities and associated deformation mechanisms is not clearly and systematically elucidated. In the specific case of intergranular fracture, one must acknowledge the role of the elastic compatibility/incompatibility and mechanisms of plastic deformation at the crack tip.

Based on geometrical and physical considerations dictating the behavior of grain boundaries both at the macroscopic and atomistic scales, this paper presents a different approach to select sets of grain boundaries when comparing their mechanical response, deconvoluting the influence of selected lattice attributes from the role of the grain boundary structure. Instead of constructing sets of grain boundary models within the misorientation/structure space by simply varying the misorientation angle around a fixed misorientation axis, sets of grain boundary models are created that sit on isocurves associated with important fracture-related attributes of the adjoining lattices. For example, for cubic materials, these lattice attributes could include the Schmid factor for primary slip, the ratio between primary and secondary Schmid factors, and the anisotropic elastic moduli of the oriented lattice regions. Other components of the stress state acting on the activated slip systems or considerations of the slip mode as necessary for hexagonal materials systems could also be chosen. The idea is to select sets of grain boundaries with multiple matching lattice attributes such that these effects are eliminated from the comparative analysis of their mechanical response. The rationale for several lattice attributes is discussed in the following section. The relevance of this approach is illustrated via atomistic simulations of intergranular fracture along a pair of Ni grain boundaries that possess matching elastic impedance and primary Schmid factor for slip. The effect of interfacial structural changes induced by segregated H at the grain boundary is used as a means to emphasize and illustrate the role of interface structure on grain boundary strength and work of separation while keeping the lattice mechanical attributes fixed in a systematic and rigorous manner. A thorough review of the role of H other than structural change is beyond the scope of this manuscript as it would detract from the main objective of the present methodology.

## Choosing grain boundary sets

Grain boundaries are classically defined using five geometric degrees of freedom^24, 25^; three degrees of freedom describe the misorientation between grains and two degrees of freedom describe the orientation of the boundary plane. However, the number of parameters required to describe the complex coupling between interfacial structure and mechanical behavior during intergranular fracture encompasses not only the five degrees of freedom describing the geometry of the grain boundary, but also the stress incompatibility controlling the deformation at the grain boundary^26^, the nature of slip activity (single slip versus multiple slip, mechanisms for slip transfer) in each neighboring crystal^27, 28^, and likely other parameters. In order to rationalize the role of the interface structure between different grain boundaries, several lattice attributes are considered to find suitable orientation couples which isolate grain boundary structure-property relationships while keeping fracture-related properties of the adjoining lattices fixed. For example, the following lattice attributes may be considered as starting selection criteria relevant for a FCC metal based on their impact on crack propagation:

1. The elastic anisotropy between the two grains.
2. The magnitude of the Schmid factor for primary slip.
3. The ratio of Schmid factors between the primary and secondary slip systems.

**Figure 1:**
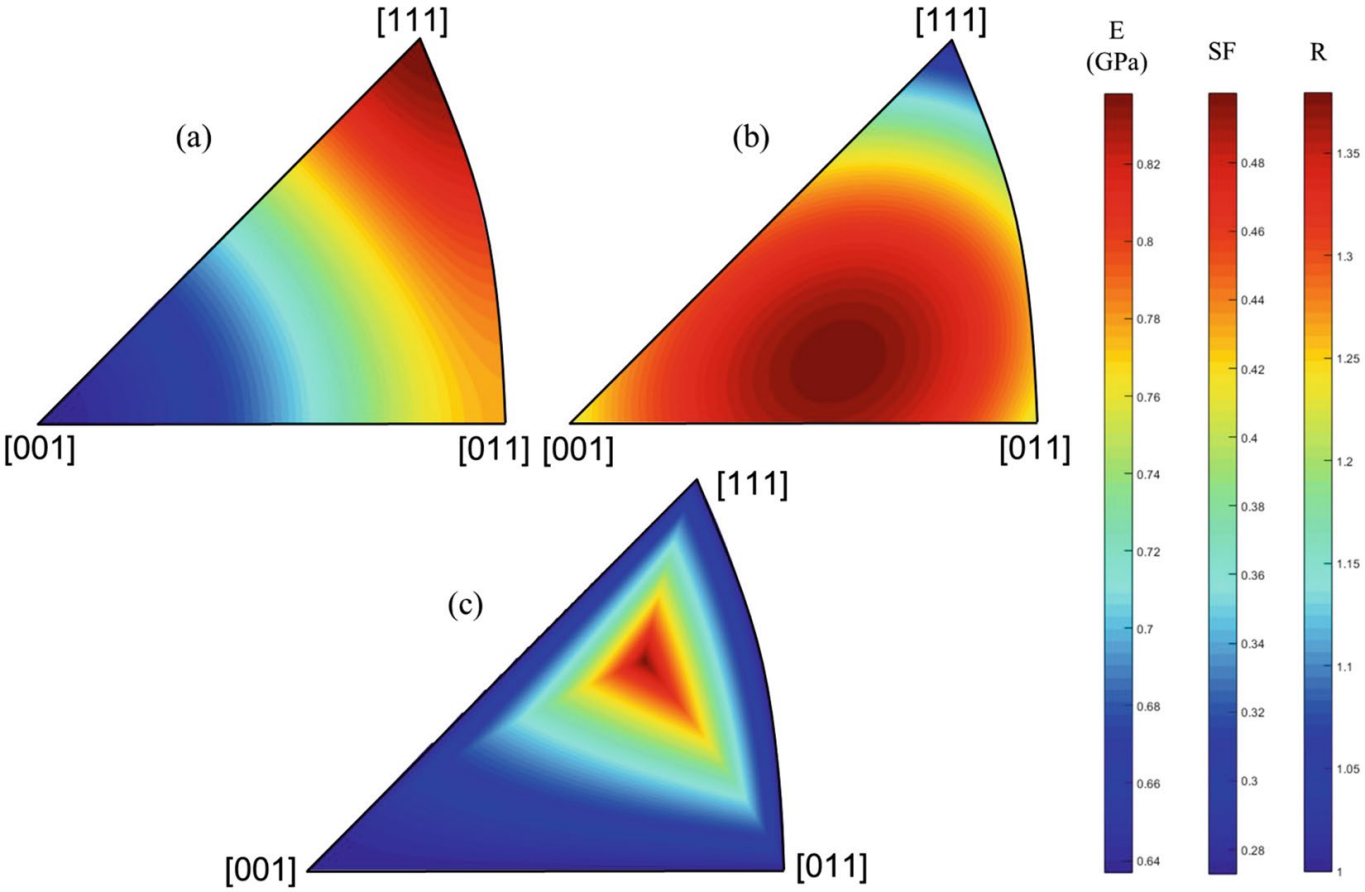
Standard stereographic triangle showing (**a**) isocurves for the directional Young’s modulus, (**b**) the maximum Schmid factor on any 〈110〉{111} slip system in a FCC crystal, and (**c**) isocurves for the ratio between the primary and secondary Schmid factor in a FCC crystal.

Within the context of fracture, it is convenient to analyze the mechanical behavior of grain boundaries with respect to a given reference axis $\overrightarrow{X}=\overrightarrow{X}({x}_{1},{x}_{2},{x}_{3})$ (as discussed later, the axis normal to the grain boundary plane is considered as the reference axis in the present work). As such, a representation of lattice properties using isocurves within the inverse pole figure along that reference direction is most appropriate. All orientations falling on the same isocurves can be physically interpreted as orientations having identical lattice properties. In the case of FCC lattices, analyses are performed in the standard stereographic triangle, as shown in Fig. 1.

## Elastic anisotropy

The first attribute considered is the elastic impedance, i.e., the directional Young’s modulus with respect to the reference axis (normal to the grain boundary in the present study). The difference in elastic anisotropy due to crystallographic misorientation is not only a cause for localization effects at the grain boundary (image forces) but also a driving factor for crack propagation (e.g., Dundurs’ parameters). The orientation dependent Young’s modulus ${E}_{\overrightarrow{X}}$ for the reference direction $\overrightarrow{X}$ within each crystal is given by^29, 30^:

1 $$\frac{1}{{E}_{\overrightarrow{X}}}=\frac{{{\mathbb{C}}}_{11}+{{\mathbb{C}}}_{12}}{({{\mathbb{C}}}_{11}-{{\mathbb{C}}}_{12})({{\mathbb{C}}}_{11}+2{{\mathbb{C}}}_{12})}+(\frac{1}{{{\mathbb{C}}}_{44}}-2\frac{1}{{{\mathbb{C}}}_{11}-{{\mathbb{C}}}_{12}})\frac{{x}_{1}^{2}{x}_{2}^{2}+{x}_{2}^{2}{x}_{3}^{2}+{x}_{1}^{2}{x}_{3}^{2}}{{\Vert \overrightarrow{X}\Vert }^{4}}\mathrm{\ ,}$$

where ${{\mathbb{C}}}_{ij}$ are the anisotropic elastic constants. Sets of grain boundary models may be constructed so that the directional Young’s modulus ${E}_{\overrightarrow{X}}$ is the same for all grain boundary models within the set. Isocurves showing the orientation dependence of the Young’s modulus are shown in Fig. 1(a) within the standard stereographic triangle in the case of nickel (${{\mathbb{C}}}_{11}=246.4$ GPa, ${{\mathbb{C}}}_{12}=147.3$ GPa, ${{\mathbb{C}}}_{44}=124.8$ GPa, from the interatomic potential used in this work^31^). The anisotropic Poisson’s ratios of the lattice with respect to the grain boundary normal could also be considered as attributes to match within a set of grain boundary models. In this work, the inclusion of this aspect of the lattice is partially satisfied via the selection of symmetric tilt grain boundaries for analysis. In the case of grain boundaries which do not present such geometrical symmetries or for reference directions that may be more complex with respect to the grain boundary plane, the effects of the full heterogeneity and compatibility of the elastic fields^26^ need to be considered when deriving an elastic anisotropy criterion for constructing grain boundary sets.

## Schmid factor for primary slip

The second attribute considered accounts for one aspect of plasticity at the crack tip. Assuming that all slip systems intersect with the crack tip, sets of grain boundary models may be constructed so that the Schmid factor for primary slip *μ* ^*α*^ on slip system *α* is identical, meaning that the largest resolved shear stress on any slip system is the same for all grain boundary models within the set. The resolved shear stress *τ* ^*α*^ is calculated as the projection of the applied stress *σ* based on the angle *χ* formed by the normal to the slip system and direction of the applied load and based on the angle *θ* formed by the glide plane and the applied loading axis such that,

2 $${\tau }^{\alpha }={\mu }^{\alpha }\cdot \sigma \,{\rm{with}}\,{\mu }^{\alpha }=\,\cos \,\chi \,\cos \,\theta .$$

Figure 1(b) shows the maximum (primary) Schmid factor within the standard stereographic triangle according to the reference direction.

## Slip mode: Single slip versus multi-slip

Acknowledging that slip mode is important, a third criterion that may be used to select sets of grain boundary models provides a description of the proximity to a multi-slip lattice orientation. If the Schmid factors are ordered (for the set of slip systems that intersect the crack tip) from maximum to minimum magnitude, the ratio *R*_*μ*_ between the two highest Schmid factors (for the same reference direction) can be used as a metric to evaluate the slip mode and the potential for a second slip system to be activated during loading. The ratio *R*_*μ*_ is defined as,

3 $${R}_{\mu }=\frac{{\mu }_{1}}{{\mu }_{2}},$$

where *μ*_1_ and *μ*_2_ are the largest (primary) and second largest (secondary) Schmid factors, respectively. A ratio *R*_*μ*_ close to unity indicates that plastic deformation will occur via activation of two or more slip systems, whereas a value of *R*_*μ*_ > 1 indicates that plastic deformation occurs via activation of a single slip system. The value of the ratio *R*_*μ*_ is shown within the stereographic triangle in Fig. 1(c). The outside edges of the triangle represent multi-slip configurations (*R*_*μ*_ = 1), where more than one slip system is simultaneously activated. Points within the interior of the triangle (*R*_*μ*_ > 1) represent single slip configurations, where one slip system has a higher Schmid factor than all others. In the case of materials with a hexagonal crystal structure, nature of the deformation mode, i.e., prismatic versus pyramidal versus basal could be instead considered as an attribute to match within a set of grain boundary models.

## Defining sets of grain boundaries

**Figure 2:**
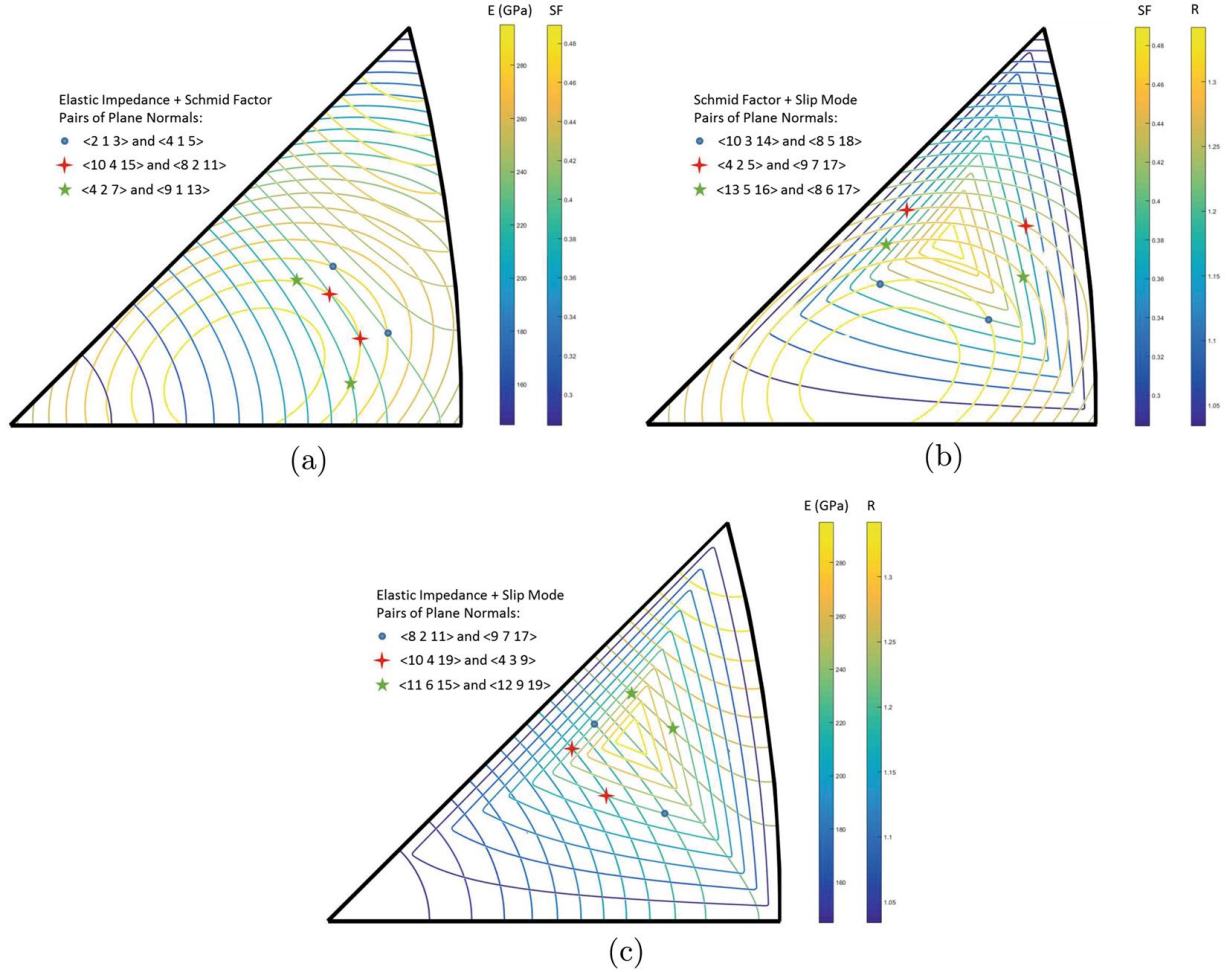
(**a**) Overlay of elastic impedance and primary Schmid factor, (**b**) overlay of primary Schmid factor and slip mode, and (**c**) overlay of elastic impedance and slip mode.

Sets of grain boundaries may be chosen such that two (or more) of the above attributes of the adjoining lattices are identical, thereby eliminating the role of these selected lattice attributes in the comparative analysis of intergranular fracture. This condition is satisfied when isocurves within the stereographic triangle associated with the specific lattice attributes intersect. This approach is shown in Fig. 2 for each combination of lattice attributes considered in the above discussion. Focusing on Fig. 2(a), many possibilities exist, with three grain boundary normal pairs identified with matching directional Young’s modulus (with respect to the loading axis) and Schmid factor for primary slip. Table 1 provides data associated with a few example orientations identified with markers in Fig. 2. From these grain boundary normals, one can create different sets of grain boundary models spanning a range of grain boundary structures, including both tilt and twist interfaces, while being mechanically matched. Note, the search for matching structures is limited to *h*, *k*, *l* < 20 for practical reasons pertaining to the construction of the periodic simulation cell in the atomistic grain boundary models, as discussed in the “*Methods*” Section. Thus, while most pairs have identical properties, small error (less than 1%) is allowed in the selection process.

**Table 1:** Example grain boundary normal pairs for comparative analysis of intergranular fracture. The two grain boundaries marked with an asterisk symbol (*) are used in the case study in this manuscript.

Matching lattice attributes	GB Normal	GB CSL	${{\boldsymbol{E}}}_{\overrightarrow{{\boldsymbol{X}}}}$ [GPa]	*μ*	*R*_*μ*_
$({E}_{\overrightarrow{X}},\mu )$	〈2 1 3〉^*^	Σ7	232.5	0.467	1.33
	〈4 1 5〉^*^	Σ21	232.5	0.467	1.20
	〈10 4 15〉	Σ341	225.0	0.478	1.25
	〈8 2 11〉	Σ189	224.6	0.477	1.17
	〈4 2 7〉	Σ69	213.9	0.479	1.25
	〈9 1 13〉	Σ251	215.0	0.478	1.07
(*μ*, *R*_*μ*_)	〈10 3 14〉	Σ305	225.7	0.477	1.20
	〈8 5 18〉	Σ413	193.3	0.477	1.20
	〈4 2 5〉	Σ45	252.9	0.444	1.17
	〈9 7 17〉	Σ419	224.0	0.444	1.17
	〈13 5 16〉	Σ450	243.6	0.457	1.17
	〈8 6 17〉	Σ389	206.9	0.458	1.16
$({E}_{\overrightarrow{X}},{R}_{\mu })$	〈8 2 11〉	Σ189	224.6	0.477	1.17
	〈9 7 17〉	Σ419	224.0	0.444	1.17
	〈10 4 19〉	Σ477	199.3	0.492	1.16
	〈4 3 9〉	Σ53	200.2	0.462	1.15
	〈11 6 15〉	Σ191	247.8	0.448	1.24
	〈12 9 19〉	Σ293	246.8	0.429	1.24

As the focus of this work is on the presentation of the grain boundary selection methodology, only the {213} and {415} grain boundaries are chosen for comparative analysis of grain boundary decohesion behavior. Their lattice attributes are listed in Table 1. Although these boundaries are selected by matching elastic impedance and maximum primary Schmid factor, it should be noted that these boundaries also have fairly close *R*_*μ*_ ratios. Furthermore, only symmetric tilt grain boundaries are constructed with a 〈111〉 misorientation axis which automatically matches one of the relevant Poisson’s ratios between the grain boundary models. With this selection, the grain boundary structure can be easily identified for each interface in terms of the classical structural unit model^32^. The $({E}_{\overrightarrow{X}},\mu )$ couple corresponds physically to the importance of the elastic-plastic transition during the crack propagation process. Other combinations of lattice attributes can also be matched to assess the importance of other physical processes during intergranular fracture. For example, grain boundaries with matching directional Young’s modulus and slip mode, i.e., matching $({E}_{\overrightarrow{X}},{R}_{\mu })$ couples, may illustrate the importance of the slip plane activation ahead of the crack tip. Grain boundaries that have matching primary Schmid factor and slip mode, i.e., matching (*μ*, *R*_*μ*_) couples, allow for an evaluation of elastic impedance mismatch effects on intergranular fracture processes.

## Comparing intergranular fracture between grain boundaries

### Comparison of grain boundary structures

**Figure 3:**
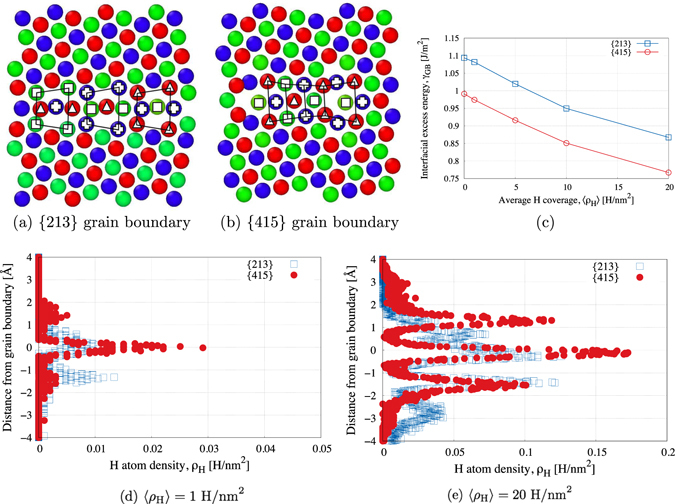
(**a**,**b**) Atomic structures for (**a**) Σ7 [111]/(213) and (**b**) Σ21 [111]/(415) symmetric tilt grain boundaries. (**c**) Grain boundary energy per unit area *γ*_*GB*_ as a function of average H coverage 〈*ρ*_*H*_〉. (**d**,**e**) H atom density *ρ*_*H*_ along the grain boundary after NPT equilibration to 300 K and the hydrostatic prestress for (**d**) 1 H/nm^2^ and (**e**) 20 H/nm^2^. The H distribution is different emphasizing the role of grain boundary structure.

Minimum energy grain boundary structures for both {213} and {415} grain boundaries are compared in Fig. 3(a,b). Atoms in Fig. 3(a,b) are colored by {111} planes. The structure of the {213} grain boundary can be described as EF.EF.EF in the structural unit model (the dot signifies that equivalent atoms in each period are relatively displaced along the tilt axis), while the structure of the {415} grain boundary can be described as EEEF’ in the structural unit model. Note that the structures observed are consistent with those characterized elsewhere^32^. The structure of these boundaries is a direct and sole consequence of the crystallography of the interface and the geometrical and energetic construct used to obtain these grain boundaries.

Since the purpose of this methodology is to isolate the role of grain boundary structure from the role of the attributes of the adjoining lattices on intergranular fracture, the addition of H at the grain boundaries is chosen as a proxy to change the boundary structure while keeping the lattice-related fracture properties fixed and emphasize its role in the identification of structure-property relationships.

One possible metric chosen to represent the change of grain boundary structure is the average number of H atoms per unit grain boundary area 〈*ρ*_*H*_〉 measured in H/nm^2^. A second metric uses the relative change of the (excess) grain boundary energy *γ*_*GB*_ upon H interfacial segregation. Figure 3(c) shows the change in *γ*_*GB*_ as a function of 〈*ρ*_*H*_〉 for {213} and {415} grain boundaries. The non-linear decrease of the grain boundary energy as the average H coverage increases illustrates the alteration of the structure of the grain boundary^33^ induced by the presence of H atoms (assuming that H atoms can be considered as misfitting spherical inclusions^34^). Such structural changes are evident by observing the lattice positions occupied by H atoms within each grain boundary at equilibrium temperature and hydrostatic pressure (before the crack is introduced) along the reference direction (i.e., direction normal to the grain boundary). Specifically, as shown in Fig. 3(d,e), for the {415} grain boundary, a high density of H atoms are positioned along the grain boundary plane (*y* = 0) and the distribution of H is relatively symmetric. However, for the {213} grain boundary, in addition to H atoms at the grain boundary plane, H atoms occupy asymmetric lattice sites on either side of the grain boundary plane. This observation is in agreement with the pristine (H-free) grain boundary structures in Fig. 3(a,b), where the {213} grain boundary has a higher density of Ni atoms along the grain boundary plane (and thus lower free volume) compared to the {415} grain boundary promoting more H segregation and thus favoring this asymmetry. Additionally, as shown later, this difference in the symmetry of the grain boundary structure with H between both boundaries will have a direct consequence on associated interfacial fracture properties.

### Comparison of tensile strength and work of separation

####  Traction-separation relationship

**Figure 4:**
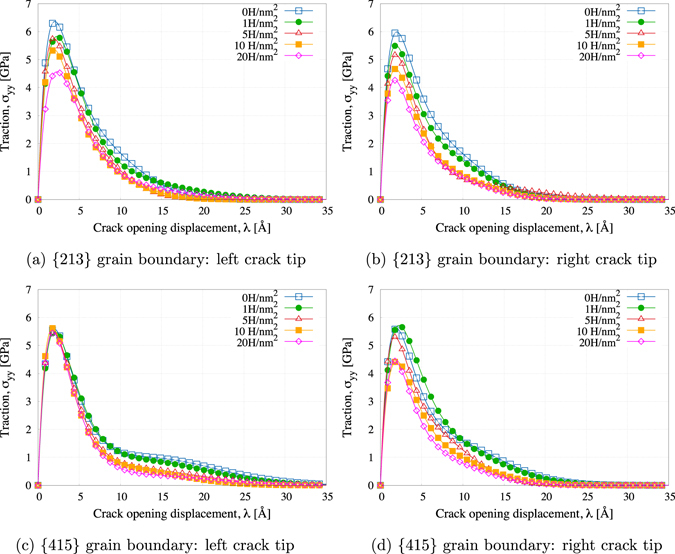
Decohesion curves for crack propagation in left/right directions for each concentration of H at the grain boundary. Curves are extracted from the density of decohesion states using the CZVE method during steady-state crack growth.

The grain boundary models constructed are mechanically equivalent in the sense that critical fracture-related lattice attributes have been matched, but, as pointed out in the previous subsection and from Fig. 3, it is clear that they have measurably different structures. Thus, a comparative analysis of their decohesion responses enables the identification of grain boundary structure-property relationships. The statistical methodology discussed in the “*Methods*” Section is employed to extract the traction-separation relationship from the density of states *ρ*(*λ*, *σ*_*yy*_) as a function of H coverage during steady-state crack growth. Three independent simulations are performed for each H coverage, with different random initializations of the thermal velocities and in the process of adding H, in order to (i) increase the number of data points in the density of decohesion states and (ii) understand the statistical variability associated with molecular dynamics simulations during the extraction of the average behavior. An analytical form for the grain boundary decohesion relationship is extracted from the density of states distribution *ρ*(*λ*, *σ*_*yy*_) by fitting the data to,

4 $${\sigma }_{yy}(\lambda )={\sigma }_{0}ez\frac{\lambda }{{\lambda }_{0}}exp(-z\frac{\lambda }{{\lambda }_{0}})+{\sigma }_{1}{[ez\frac{\lambda }{{\delta }_{1}}]}^{2}\exp (-{[z\frac{\lambda }{{\lambda }_{1}}]}^{2}),$$

where (*σ*_0_, *σ*_1_, *λ*_0_, *λ*_1_) are the four fitting parameters used, *z* = 16*e*/9 and *e* = exp(1). The definition of the traction-separation relationship in Eq. (4) is motivated by a combination of an exponential-based traction-separation relationship (first part of Eq. (4)) which appropriately captures the short range decohesion behavior, with another exponential-based functional form (second part of Eq. (4)) meant to capture the crack tip opening behavior for large crack openings (more ductile behavior). Note that this functional form satisfies the generic required characteristics for cohesive constitutive relationships^35^: (i) the work to create a new surface is finite, and its value corresponds to the work of separation, (ii) the cohesive traction across the fracture surface generally decreases to zero while the separation increases under the softening condition, and (iii) a potential for the cohesive constitutive relationship may exist, thus making the energy dissipation associated with unloading/reloading independent of a potential. Figure 4 shows the traction-separation curve fits using Eq. (4) for positive (right) and negative (left) direction crack growth along the {213} and {415} grain boundaries for each H coverage considered. A conventional non-linear least-squares (NLLS) Marquardt-Levenberg algorithm is used to fit *σ*_*yy*_(*λ*) to *ρ*(*λ*, *σ*_*yy*_). The complete set of data for the intergranular fracture simulations for both grain boundaries and all realizations is provided in the Supplemental documentation.

For each grain boundary and each crack propagation direction, two fundamental quantities characterize the traction-separation relationships, namely the work of separation *γ*_*f*_  and the grain boundary tensile strength *σ*_max_ (also referred to as peak tensile stress throughout the text). The work of separation is given by^36, 37^

5 $${\gamma }_{f}=2{\gamma }_{s}-{\gamma }_{GB}+{\gamma }_{p}={\int }_{0}^{\infty }{\sigma }_{yy}(\lambda )d\lambda ,$$

where *γ*_*s*_ and *γ*_*p*_ are the surface energy (free surface) and the energy associated with the plastic work per unit area, respectively. The peak tensile stress *σ*_max_ is taken as the maximum cohesive stress reached during the separation process (i.e., between zero and a sufficiently large crack opening displacement *λ*_max_).

**Figure 5:**
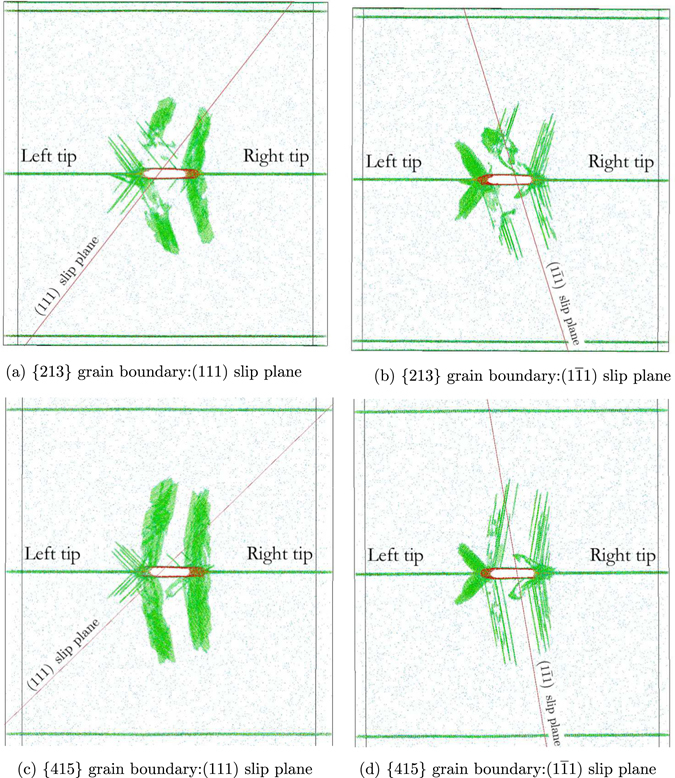
Dislocation activity along the (111) and (1$\overline{1}$1) slip planes during crack propagation for the {213} and {415} grain boundaries for an average H coverage 〈*ρ*_*H*_〉 = 10 *H*/*nm* ^2^ at 10 ps. Atoms are colored by centrosymmetry parameter.

Figure 5(a–d) shows dislocation nucleation during crack propagation from left/right crack tips for the {213} and {415} grain boundary models. Atoms with a centrosymmetry value greater than 2.0 *Å* ^2^ are omitted from the images. The images are oriented so that the activated slip planes can be clearly identified. The orientation of the slip plane of interest is identified in Grain 1 (see nomenclature defined in Fig. 7); since symmetric tilt grain boundary models are studied, the mirror image of this identified slip plane is activated in Grain 2.

Figure 5 shows that two slip planes are consistently activated during crack propagation. The $(1\overline{1}1)$ plane is the slip plane associated with the primary slip system (maximum Schmid factor). The (111) slip plane is also activated for both {213} and {415} models due to the direction of crack propagation relative to the lattice orientation. Qualitatively, dislocation activity is similar for {213} and {415} grain boundary models. Thus, the plastic work associated with *γ*_*f*_  is concluded to be consistent between {213} and {415} grain boundary models, confirming the approach used to select this pair of grain boundaries for analysis. Therefore, differences in tensile strength and work of separation, and their dependence on H coverage, is attributed to differences in interfacial structure.

**Figure 6:**
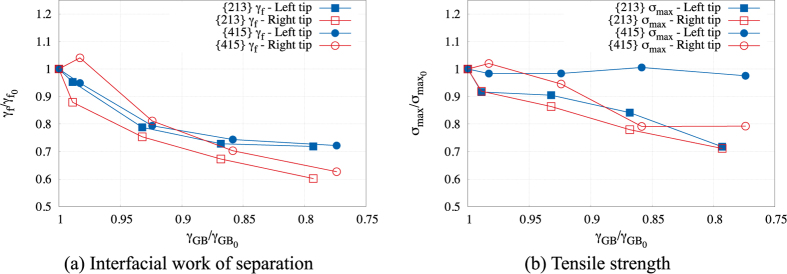
Relative change of the work of separation *γ*_*f*_ and the grain boundary tensile strength *σ*_max_ extracted from the density of decohesion states *ρ*(*σ*_*y*__*y*_, *λ*) as a function of the relative change of grain boundary energy *γ*_*GB*_ for both grain boundaries. Each data point considers data from three independent simulations of intergranular fracture.

Figure 6(a,b) shows the relative change of the work of separation and maximum tensile stress for both grain boundaries as a function of the relative change in grain boundary energy, i.e., as a function of the change in grain boundary structure. The work of separation and maximum tensile stress data are normalized by their H-free values for the corresponding crack propagation direction. The non-normalized data is available in the Supplemental documentation.

#### Work of separation

It is notable that, for both grain boundaries and crack propagation directions, the work of separation *γ*_*f*_  shows a non-linear dependence on the grain boundary energy. In light of Eq. (5), such non-linearity clearly draws attention to the competition between the energetically driven and structurally driven dependence on the work of separation (a linear dependence would indicate an energetically driven process only). Comparing the left and right crack tips, it should be noted that the directionality of the structural units with respect to the crack propagation directions also has an impact on the degree of sensitivity of the work of separation to the structural change. Indeed, as seen in Fig. 6(a), the work of separation associated with the left crack tips (crack propagation in the negative direction) is generally more sensitive to the grain boundary structure compared to the right crack tips (crack propagation in the positive direction), especially at the highest levels of H coverage studied.

#### Grain boundary tensile strength

Conversely, as shown in Fig. 6(b), the grain boundary tensile strength *σ*_max_ presents a different type of structure-property dependence which differs based on the grain boundary considered. Indeed, the {415} grain boundary exhibits an asymmetry in its dependence on the tensile strength to the change of grain structure based on the crack propagation direction considered, while for the {213} grain boundary, both crack tips display a similar dependence to the structural change and therefore crack propagation is similar in both directions. The tensile strength associated with the left crack tip of the {415} grain boundary remains insensitive to the change of the grain boundary structure, while the peak tensile stress associated with the right crack tip (negative crack propagation direction) decreases as the grain boundary structure changes. In other words, the applied stress required to overcome the tensile strength for the crack to propagate in the negative direction is entirely and solely determined by the mechanical attributes of the adjoining lattices, while the crack propagation in the positive direction also depends on the atomic structure as the crack unzips the structural units along the grain boundary. Although such asymmetry in the crack propagation has been recognized previously^11, 14, 18^, such observations point to the fact that the relationship between the grain boundary structure and the tensile strength is clearly the result of the competition between the grain boundary mechanical attributes of the adjoining lattices and its atomic structure depending on the direction of crack propagation.

## Summary and conclusions

This manuscript proposes a methodology to isolate the influence of grain boundary structure on fracture-related properties, namely the tensile strength and the work of separation of grain boundaries using atomistic simulations. Instead of constructing sets of grain boundary models within the misorientation/structure space by varying the misorientation angle between bounds around a fixed misorientation axis, sets of grain boundaries for comparison of their mechanical properties are created by means of isocurves associated with important fracture-related properties of the adjoining lattices. Several lattice properties are proposed, and two grain boundaries with matching elastic stiffness (normal to the grain boundary) and Schmid factor for primary slip are selected for comparison as an illustration of the selection methodology.

The decohesion response of each grain boundary is computed via a cohesive zone volume element approach, which uses a density of (*λ*, *σ*_*yy*_) states to provide a meaningful measure of the tensile strength and work of separation of a grain boundary. Statistical uncertainty between molecular dynamics simulations is not found to significantly influence the distribution of (*λ*, *σ*_*yy*_) states. Through this approach, for both grain boundaries, the structure-work of separation relationship is identified as being the result of competing effects between energetics and structural aspects. On the other hand, the structure-tensile strength relationship is recognized to be associated with coupling between the grain boundary mechanical attributes of the adjoining lattices and its structure.

Both the grain boundary selection approach and the cohesive zone volume element approach to extract a density of (*λ*, *σ*_*yy*_) states could be used for various geometries and simulation setups. The example used here is merely a demonstration of the proposed method. In other words, the grain boundary selection methodology presented in this manuscript is largely independent of the means by which fracture simulations are conducted. Usage of this method could be extended to cylindrical or spherical geometries, different ensembles, and boundary conditions. Overall, this methodology is a significant departure from the current conventional approach taken to study grain boundary using atomistic simulations. It is expected that the insights from such approach will play a critical role in how phase and grain boundaries should be modeled and understood.

## Methods

The atomistic simulation code LAMMPS (Large-scale Atomic/Molecular Massively Parallel Simulator^38^) is used to study the role of grain boundary structure on the grain boundary traction-separation relationship during steady-state crack growth. In this work, the embedded atom method (EAM) potential of Angelo and Baskes^31, 39^ is employed to describe interatomic interactions. The simulation technique is composed of three steps: (i) creation of the grain boundary, (ii) introduction and propagation of an atomically sharp crack, and (iii) data mining to extract the tensile strength and work of separation of the grain boundary. Most of the details pertaining to the calculation of steady-state intergranular crack propagation have been described elsewhere^14^.

### Equilibrium grain boundary structure

The grain boundary structure and energy are calculated using an automated approach where, for bicrystal simulation cell with three-dimensional (3D) periodic boundary conditions and a constant number of atoms, one grain is incrementally translated relative to the other, sampling a large number of initial positions, and subjected to energy minimization at each translation under stress-free boundary conditions. The translation corresponding to the minimum energy grain boundary structure after energy minimization is selected as the grain boundary structure at 0 K.

### Fracture simulation

**Figure 7:**
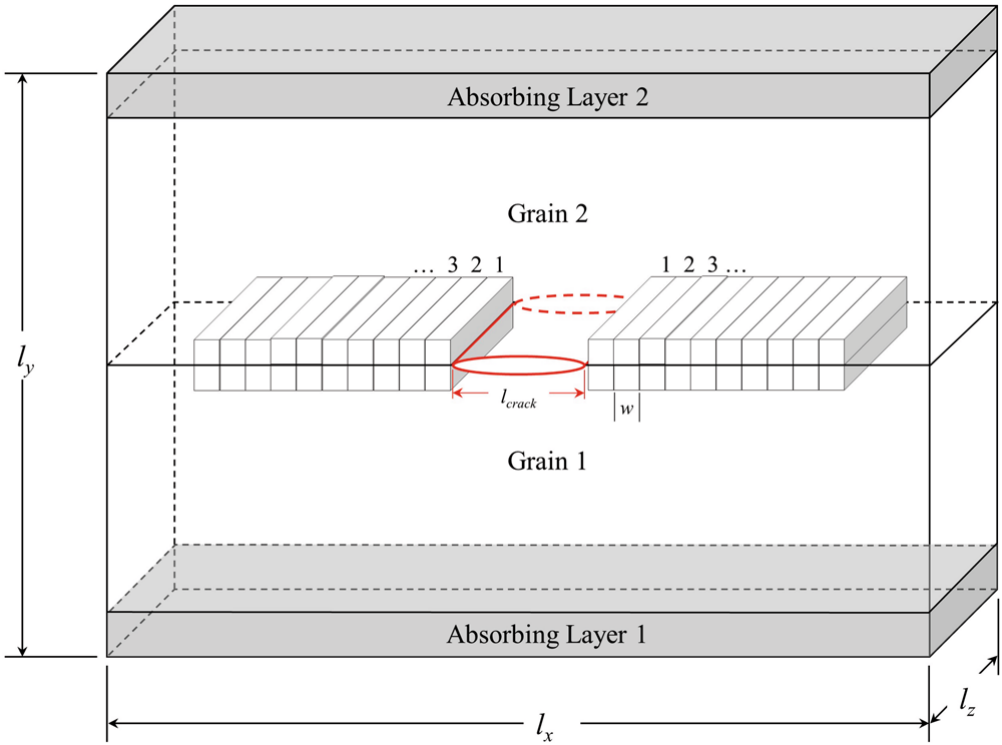
Steady state crack propagation model showing the initial crack size and the definition of the cohesive zone volume elements. This model is adapted from that of Yamakov *et al*.^11, 40^ and used in work by Barrows *et al*.^14^.

Figure 7 shows a schematic of the intergranular fracture model implemented in this work. The model consists of a primary grain boundary between Grain 1 and Grain 2, and absorbing layers whose purpose is to block dislocations from propagating away from the crack tip through the periodic boundary in the Y-direction. The dimensions of the simulation cell are approximately 0.1 *μ*m in the X- and Y-directions, and 0.01 *μ*m in the Z-direction, resulting in approximately 9 million atoms in each atomistic model. This simulation model size is sufficient so that the crack can propagate more than five times its initial length without significant self-image force interactions through the periodic boundaries.

After the intergranular fracture model is created, the ensemble is equilibrated to a temperature of 300 K under a hydrostatic tension *σ*_*h*_ = 8.0 GPa for 25 ps using isobaric-isothermal (NPT) boundary conditions. As a result, the model is strained to provide a sufficient driving force for crack propagation (once the crack is created). Once the system is thermodynamically equilibrated, the simulation is switched to the isovolume-isothermal (NVT) ensemble, preserving the simulation cell dimensions associated with the hydrostatic prestress. Following a brief additional equilibration, an atomically sharp crack of length *l*_*crack*_ = 150 Å is introduced in the center of the primary grain boundary, as shown in Fig. 7. The crack is created by screening the interatomic interactions between atoms in Grain 1 and Grain 2 along *l*_*crack*_ through the thickness of the model. This crack geometry does not consider crack front curvature effects. With this crack geometry, all FCC slip systems may intersect with the advancing crack tip, which may not be the case in truly 3D crack geometries. Thus, the use of maximum Schmid factor and the ratio of maximum Schmid factors has meaning in the selection of the grain boundaries. However, for different shape cracks such as penny-shaped cracks^41^ this may not be true and additional constraints should be applied to consider Schmid factors on slip systems with geometric intersection with the advancing crack. With the imposed strain due to the prestress *σ*_*h*_ and initial crack *l*_*crack*_, the system is evolved for 35 ps which allows both crack tips (left and right directions) to propagate along the grain boundary.

The peak stress and work of separation for the grain boundary are extracted from atomic forces and displacements ahead of each crack tip by tracking the evolution of a set of cohesive zone volume elements (CZVEs) during steady-state crack propagation. Specifically, before the atomically sharp crack is introduced, each atom within a distance *h* on either side of the primary grain boundary is assigned to a three-dimensional rectangular volume element (CZVE), as shown in Fig. 7. The bounds of the CZVEs are determined by partitioning the prestressed system near the grain boundary into 4*n* zones with 2*n* zones above and 2*n* zones below the primary grain boundary, resulting in *n* CZVE pairs along the left and right crack propagation directions. Cohesive zones above and below the initial 150 Å crack are omitted and not used to determine the tensile strength and work of separation of the grain boundary.

The state of each CZVE at position a X-position along the grain boundary and time *t* is defined by two state variables: (i) the average normal stress, *σ*_*yy*_(*X*, *t*), computed using the virial stress definition, and (ii) the crack tip opening displacement, *λ*(*X*, *t*), computed by tracking the change in the Y-position of the center of mass of two vertically neighboring cohesive zones (normal to the grain boundary plane) as the crack propagates. Thus, each CZVE state is represented as a point (*λ*, *σ*_*yy*_) at every snapshot in time. Using a statistical mechanics approach, in the limit of steady-state crack propagation that occurs over an infinitely long time over an infinitely long interface, all realized CZVE states will produce a density of states *ρ*(*λ*, *σ*_*yy*_) that is a continuous function independent of time. A functional form may be fit to the data that represents the time-independent decohesion response of the grain boundary. The *ρ*(*λ*, *σ*_*yy*_) data is filtered to only include data during steady-state crack growth. Furthermore, CZVEs that are elastically deformed far ahead of the crack tip are removed from the data^14^. The complete set of data for all realizations of all intergranular fracture simulations is provided in the Supplemental documentation.

### Adding H to the grain boundary

Simulations of intergranular fracture are performed with and without segregated H at the grain boundary. To model H segregation to the grain boundary, H atoms are randomly added to the grain boundary region in small batches, followed by an energy minimization of the grain boundary structure. The energy of each H atom is computed after energy minimization, and H atoms with energies that are greater than −0.43 eV are removed^14^ as they are deemed to be in non energetically favorable positions within the grain boundary. This process is repeated until the desired H coverage at the grain boundary is achieved.

The (excess) grain boundary energy *γ*_*GB*_ per unit area is determined classically,

6 $${\gamma }_{GB}=\frac{1}{A}[\sum _{{N}_{{\rm{Ni}}}}({E}_{{N}_{{\rm{Ni}}}}-{E}_{{N}_{{\rm{Ni}}}}^{{\rm{bulk}}})+\sum _{{N}_{{\rm{H}}}}({E}_{{N}_{{\rm{H}}}}-{E}_{{N}_{{\rm{H}}}}^{{\rm{bulk}}})]\mathrm{\ ,}$$

where *A* is the surface area of the grain boundary, *N*_*Ni*_ and *N*_*H*_ are the total number of Ni and H atoms within the system respectively, ${E}_{{N}_{{\rm{Ni}}}}$ and ${E}_{{N}_{{\rm{H}}}}$ are the energy of an individual Ni and H atom within the system respectively, while ${E}_{{N}_{{\rm{Ni}}}}^{{\rm{bulk}}}$ is the bulk energy of a Ni atom (−4.45 eV), and ${E}_{{N}_{{\rm{H}}}}^{{\rm{bulk}}}$ is the energy of free H atoms (−2.31 eV)^42^.

## Electronic supplementary material

Supplemental information
