# Supplementary material for: A primer on selecting grain boundary sets for comparison of interfacial fracture properties in molecular dynamics simulations
Source: Sci Rep. 2017 Aug 21;7:8332. doi: 10.1038/s41598-017-08637-z (PMC5566553; doi:10.1038/s41598-017-08637-z)
Supplement: Supplementary file 1 — Supplemental information [file 41598_2017_8637_MOESM1_ESM.pdf]

Supplemental information for:
A primer on selecting grain boundary sets for comparison of interfacial fracture properties in molecular dynamics simulations

Rémi Dingreville, Doruk Aksoy, Douglas E. Spearot

Table 1: Complete set of fitting parameters ($\sigma_0, \sigma_1, \lambda_0, \lambda_1$) for Eq. (4) for both grain boundaries and associated crack tips.

GB Normal (crack tip)	H coverage [H/nm^2]	σ_0 [GPa]	λ_0 [Å]	σ_1 [GPa]	λ_1 [Å]
⟨2 1 3⟩ (left)	0.0	6.19	1.93	0.45	8.56
	1.0	5.79	2.26	0.18	12.41
	5.0	5.59	1.81	0.35	7.13
	10.0	5.27	1.89	0.21	8.24
	20.0	4.53	2.28	0.11	12.25
⟨2 1 3⟩ (right)	0.0	5.82	1.87	0.48	8.58
	1.0	5.37	1.79	0.41	8.62
	5.0	5.15	1.85	0.16	12.4
	10.0	4.59	1.77	0.24	8.65
	20.0	4.18	1.68	0.25	8.29
⟨4 1 5⟩ (left)	0.0	5.54	1.94	0.34	14.36
	1.0	5.45	2.01	0.30	13.78
	5.0	5.47	1.86	0.19	13.1
	10.0	5.59	1.75	0.19	11.44
	20.0	5.44	1.83	0.12	14.17
⟨4 1 5⟩ (right)	0.0	5.50	1.86	.45	10.23
	1.0	5.61	2.18	0.33	10.43
	5.0	5.14	1.64	0.46	7.72
	10.0	4.35	1.89	0.26	9.10
	20.0	4.37	1.70	0.23	8.66

Table 2: Grain boundary energy (γ_{GB}) (see Eq. (6)), fracture toughness (γ_f) (see Eq. (5)) and tensile strength ($\sigma_{\max}$) as a function of the average number of H atoms per unit grain boundary area $\langle\rho_H\rangle$ for both grain boundaries and associated crack tips.

GB Normal (crack tip)	$\langle\rho_H\rangle$ [H/nm^2]	γ_{GB} [J/m^2]	γ_f [J/m^2]	$\sigma_{\max}$ [GPa]
⟨2 1 3⟩ (left)	0.0	1.0938	4.5206	6.3621
	1.0	1.0816	4.3070	5.8307
	5.0	1.0196	3.5614	5.7570
	10.0	0.9496	3.2922	5.3538
	20.0	0.8673	3.2474	4.5648
⟨2 1 3⟩ (right)	0.0	1.0938	4.3015	5.9880
	1.0	1.0816	3.7804	5.5039
	5.0	1.0196	3.2407	5.1721
	10.0	0.9496	2.8937	4.6683
	20.0	0.8673	2.5888	4.2601
⟨4 1 5⟩ (left)	0.0	0.9912	4.5139	5.5868
	1.0	0.9742	4.2826	5.4951
	5.0	0.9159	3.5824	5.4972
	10.0	0.8509	3.3566	5.6181
	20.0	0.7671	3.2578	5.4500
⟨4 1 5⟩ (right)	0.0	0.9912	4.2696	5.6065
	1.0	0.9742	4.4424	5.7189
	5.0	0.9159	3.4634	5.3007
	10.0	0.8509	3.0008	4.4370
	20.0	0.7671	2.6759	4.4426

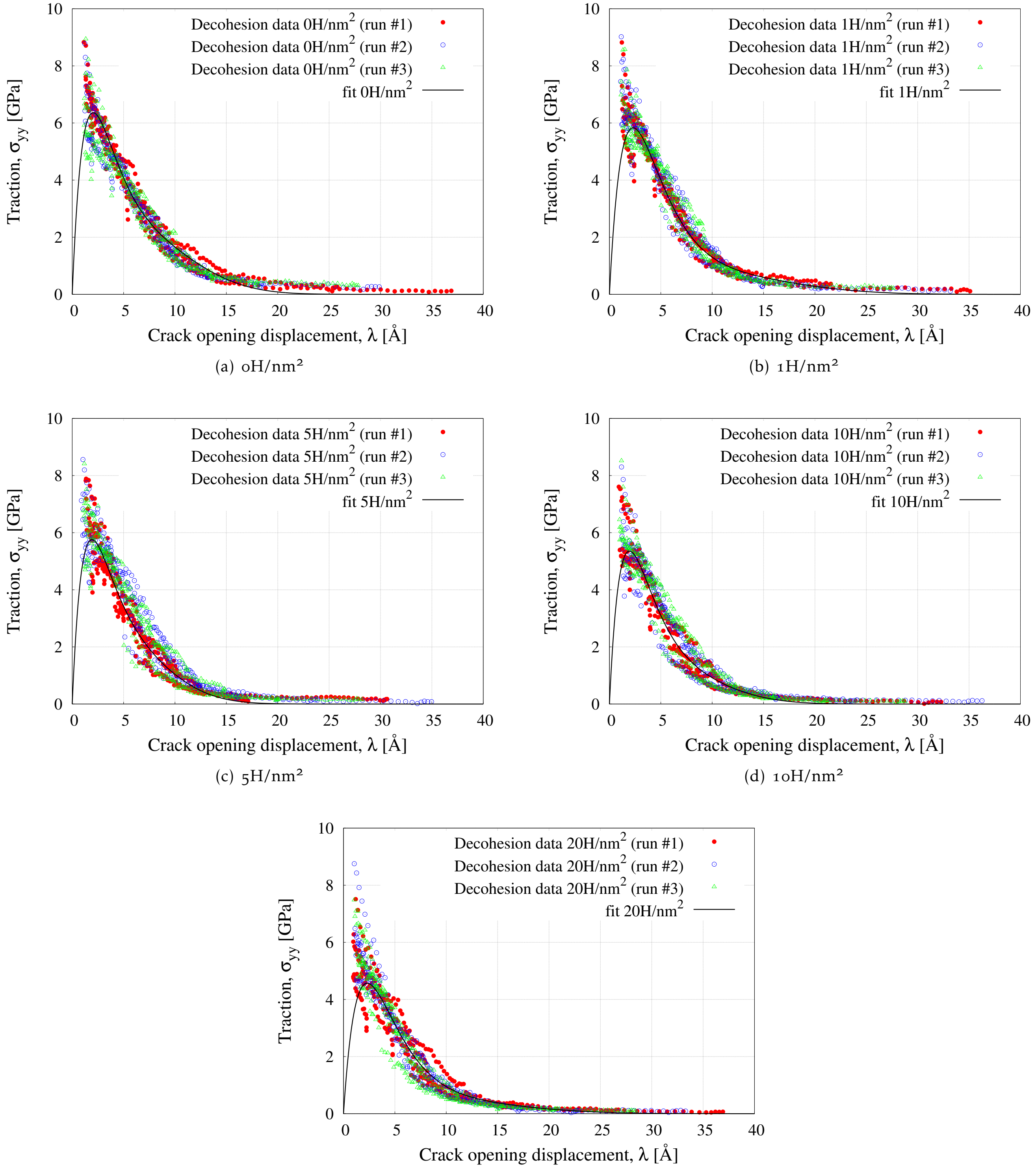

(a) 0H/nm²

(b) 1H/nm²

(c) 5H/nm²

(d) 10H/nm²

(e) 20H/nm²

Figure 1: Density of decohesion states and fitted decohesion curve for crack propagation in the negative (left) direction along the {213} grain boundary for various H coverages $\langle \rho_H \rangle$. Cohesive zone volume element data corresponding to each of the three independent simulations are shown with different markers.

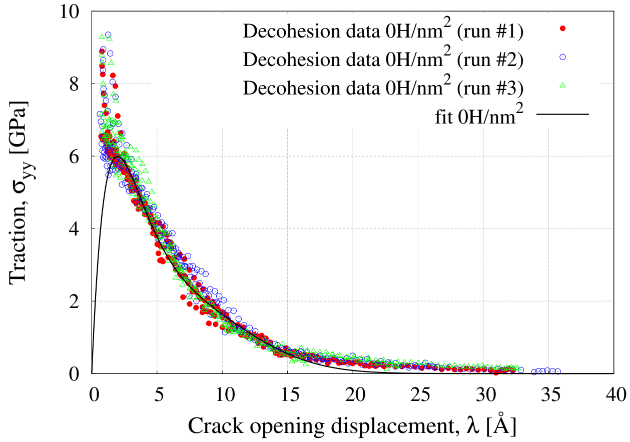

(a) 0H/nm²

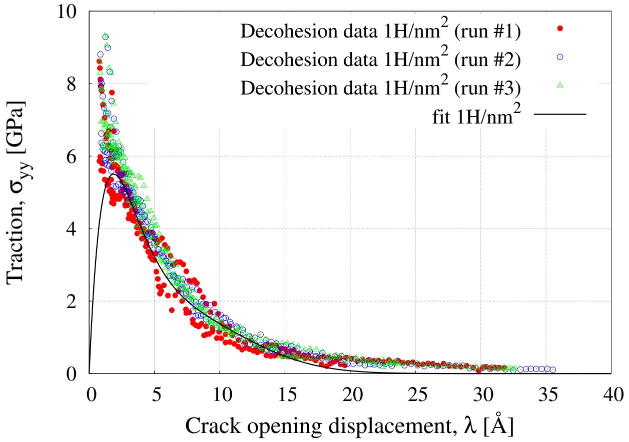

(b) 1H/nm²

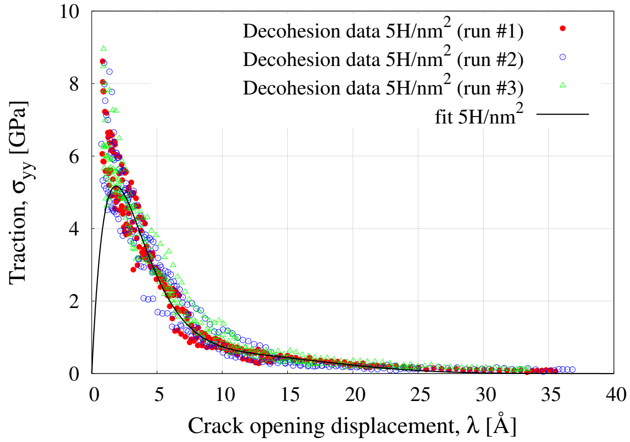

(c) 5H/nm²

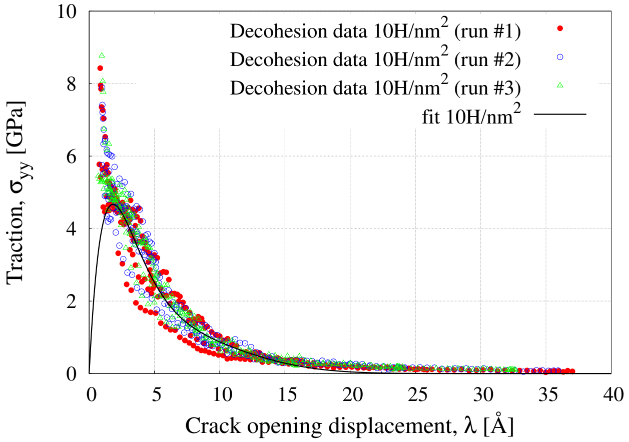

(d) 10H/nm²

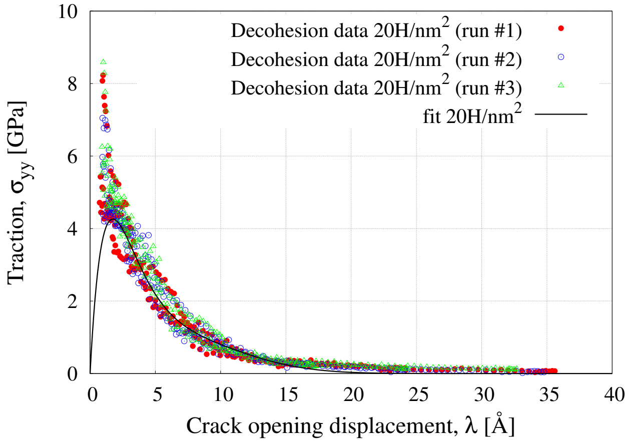

(e) 20H/nm²

Figure 2: Density of decohesion states and fitted decohesion curve for crack propagation in the positive (right) direction along the {213} grain boundary for various H coverages $\langle \rho_H \rangle$. Cohesive zone volume element data corresponding to each of the three independent simulations are shown with different markers.

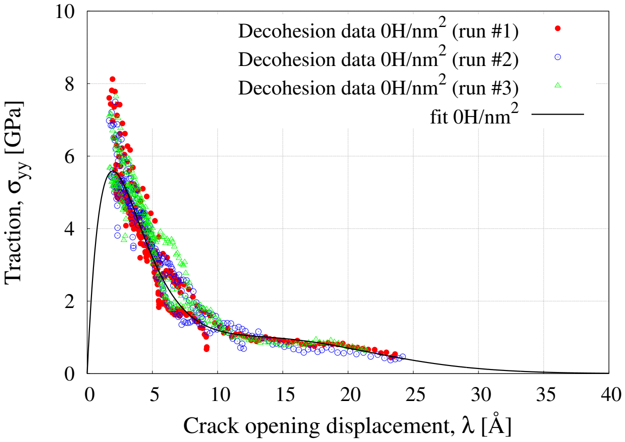

(a) 0H/nm²

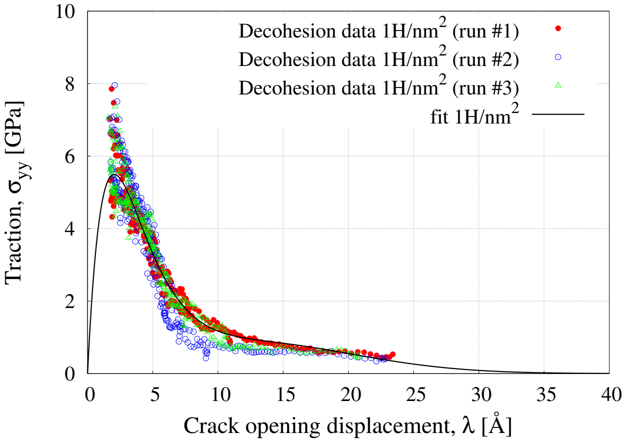

(b) 1H/nm²

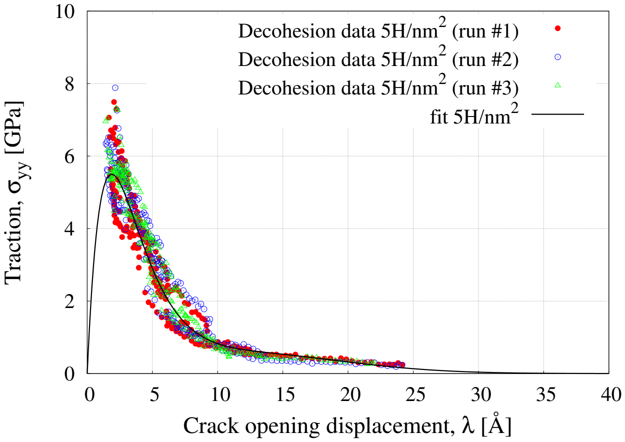

(c) 5H/nm²

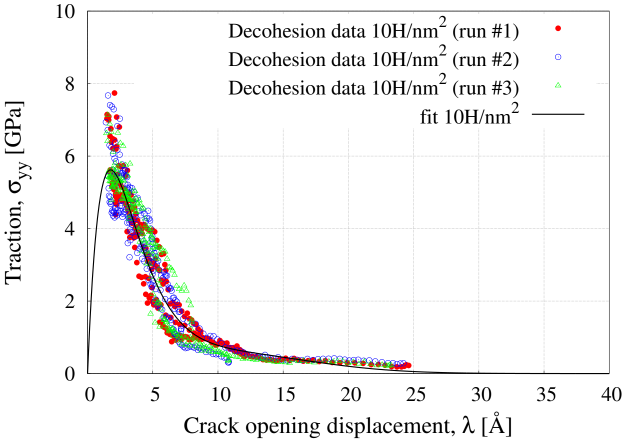

(d) 10H/nm²

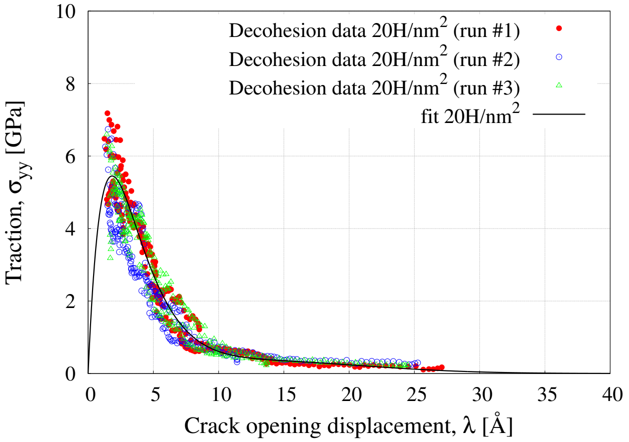

(e) 20H/nm²

Figure 3: Density of decohesion states and fitted decohesion curve for crack propagation in the negative (left) direction along the {415} grain boundary for various H coverages $\langle \rho_H \rangle$. Cohesive zone volume element data corresponding to each of the three independent simulations are shown with different markers.

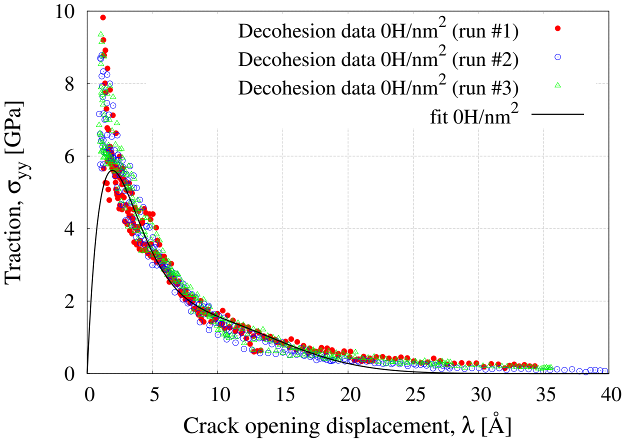

(a) 0H/nm²

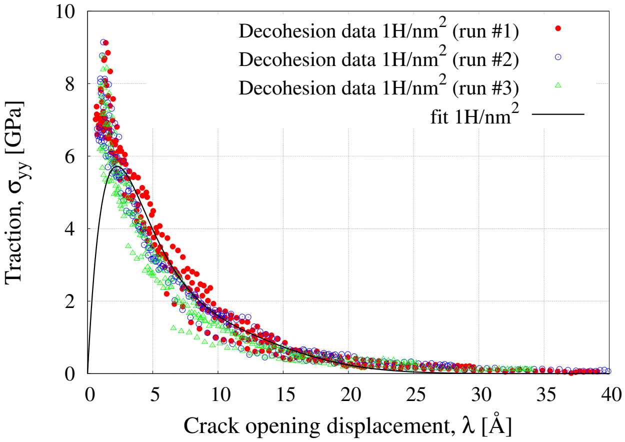

(b) 1H/nm²

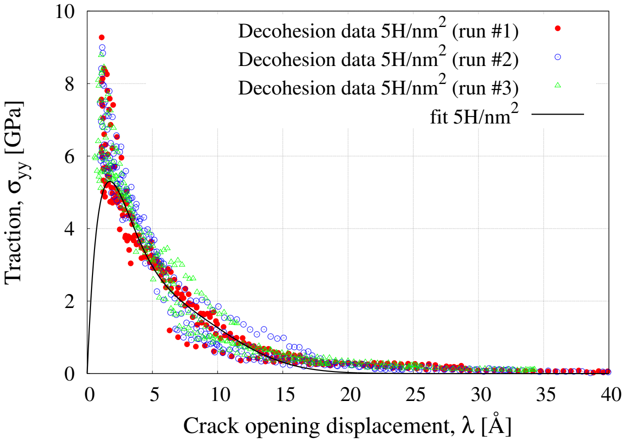

(c) 5H/nm²

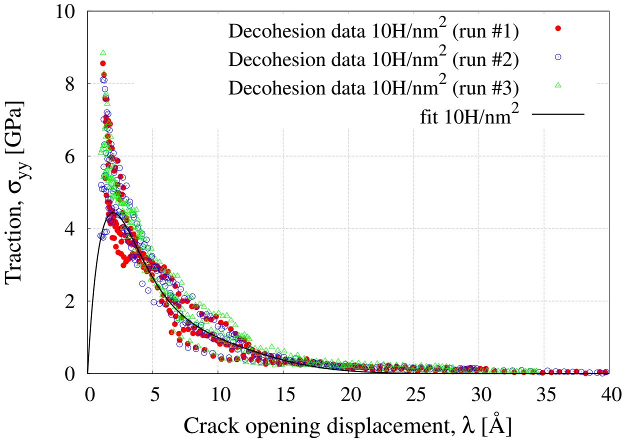

(d) 10H/nm²

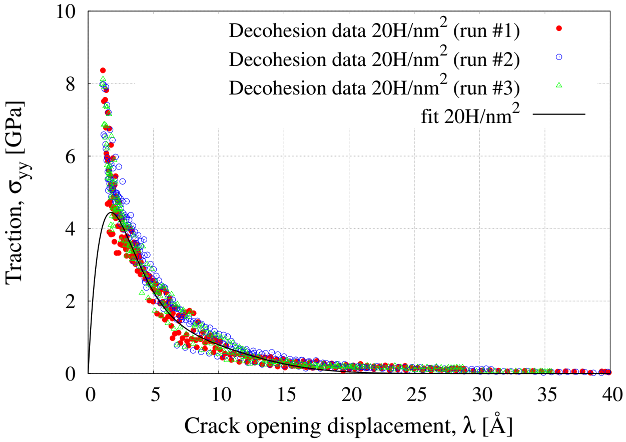

(e) 20H/nm²

Figure 4: Density of decohesion states and fitted decohesion curve for crack propagation in the positive (right) direction along the {415} grain boundary for various H coverages $\langle \rho_H \rangle$. Cohesive zone volume element data corresponding to each of the three independent simulations are shown with different markers.
